# Membrane Distribution of the *Pseudomonas* Quinolone Signal Modulates Outer Membrane Vesicle Production in *Pseudomonas aeruginosa*

**DOI:** 10.1128/mBio.01034-17

**Published:** 2017-08-08

**Authors:** Catalina Florez, Julie E. Raab, Adam C. Cooke, Jeffrey W. Schertzer

**Affiliations:** Department of Biological Sciences, Binghamton University, Binghamton, New York, USA, and Binghamton Biofilm Research Center, Binghamton University, Binghamton, New York, USA; East Carolina University; Harvard Medical School

**Keywords:** outer membrane vesicles, PQS, *Pseudomonas aeruginosa*, quorum sensing, secretion systems

## Abstract

The *Pseudomonas* quinolone signal (PQS) is an important quorum-sensing molecule in *Pseudomonas aeruginosa* that also mediates its own packaging and transport by stimulating outer membrane vesicle (OMV) formation. Because OMVs have been implicated in many virulence-associated behaviors, it is critical that we understand how they are formed. Our group proposed the bilayer-couple model for OMV biogenesis, where PQS intercalates into the outer membrane, causing expansion of the outer leaflet and consequently inducing curvature. In accordance with the model, we hypothesized that PQS must be transported from the cytoplasm to the outer membrane before it can initiate OMV formation. We initially examined two laboratory strains of *P. aeruginosa* and found significant strain-dependent differences. PQS export correlated strongly with OMV production, even though equivalent amounts of total PQS were produced by both strains. Interestingly, we discovered that poor OMV producers sequestered the majority of PQS in the inner membrane, which appeared to be the result of early saturation of the export pathway. Further analysis showed that strain-specific PQS export and OMV biogenesis patterns were stable once established but could be significantly altered by changing the growth medium. Finally, we demonstrated that the associations described for laboratory strains also held for three clinical strains. These results suggest that factors controlling the export of PQS dictate OMV biogenesis. This work provides new insight into PQS-controlled virulence in *P. aeruginosa* and provides important tools to further study signal export and OMV biogenesis.

## IMPORTANCE

Bacterial secretion has been recognized as an essential facet of microbial pathogenesis and human disease. Numerous virulence factors have been found to be transported within outer membrane vesicles (OMVs), and delivery using these biological nanoparticles often results in increased potency. OMV biogenesis is an important but poorly understood process that is ubiquitous among Gram-negative organisms. Our group seeks to understand the biochemical mechanisms behind the formation of OMVs and has developed a model of small-molecule-induced membrane curvature as an important driver of this process. With this work, we demonstrate that PQS, a known small-molecule OMV inducer, must be exported to promote OMV biogenesis in both lab-adapted and clinical strains of *Pseudomonas aeruginosa*. In supporting and expanding the bilayer-couple model of OMV biogenesis, the current work lays the groundwork for studying environmental and genetic factors that modulate OMV production and, consequently, the packaging and delivery of many bacterial factors.

## INTRODUCTION

Pathogenic bacteria make use of many strategies to deliver virulence factors to target cells. There is increasing interest in a long-known but understudied mechanism for such delivery: transport in outer membrane vesicles (OMVs). OMVs are small (50- to 300-nm) unilamellar structures that bud off from the surfaces of Gram-negative bacteria and diffuse through the environment (1). They are capable of fusing with and delivering cargo to both competing bacteria and host cells (2–5). The lipid composition and organization of OMVs resemble that of the parent outer membrane (OM) (1, 6–8). In general, this is also true of vesicular protein content, though selective packaging has been described (9–14). Cargo reportedly trafficked in this way includes virulence factors, communication signals, and nucleic acids (15–19). Accordingly, OMVs have been implicated in the avoidance of phage and immune system effectors (9, 20), interference with cytokine production and the mounting of an effective immune response (21, 22), packaging and delivery of toxins to target cells (15, 23), horizontal gene transfer mediating antibiotic resistance (4, 18), and trafficking of small-molecule communication signals (16). Because of their ability to package and transport such varied cargo, OMVs have been described as a dedicated secretion system (1, 17). They have also attracted interest as vaccine agents (24–26), drug delivery vehicles (27), and even as conceptually new antibiotics (23). Despite the important roles that OMVs are known to play in many aspects of bacterial physiology, we understand comparatively little about how they are produced. A comprehensive understanding of OMV biogenesis is much needed from the perspective of both fundamental and applied sciences.

The *Pseudomonas* quinolone signal (2-heptyl-3-hydroxy-4-quinolone; PQS) is a quorum-sensing molecule produced by *Pseudomonas aeruginosa* that is trafficked within the organism’s OMVs. PQS has also been shown to be important in stimulating the production of the same vesicles into which it is packaged (16, 28). PQS biosynthetic mutants produce markedly reduced numbers of OMVs (16), especially later in the growth phase, when PQS would normally be present (29, 30). In addition, exogenous PQS addition was shown to restore OMV production both in a mutant lacking the PQS receptor and in PQS-null cells in which protein synthesis was inhibited by antibiotic treatment (16). These findings indicate that PQS-induced OMV biogenesis does not function through a signaling mechanism or through the induction of a cascade involving *de novo* protein synthesis. Both the PAO1 (31) and PA14 (32) strains display reduced OMV production when grown under conditions of diminished oxygen concentration. Molecular oxygen is a substrate required for PQS production (32), further supporting the notion that OMV production is significantly diminished in the absence of PQS.

From these initial observations, we developed an OMV biogenesis model based upon the biophysical effects of PQS on the OM. The bilayer-couple model describes the accumulation of PQS in the outer leaflet of the OM to the extent that this leaflet is laterally expanded relative to the inner leaflet. Relaxation of this interleaflet tension through the induction of membrane curvature is then proposed to provide the initial driving force for OMV formation (33). The strong interactions that PQS has with the lipid A portion of lipopolysaccharide (LPS) (34) and the sensitivity and specificity of the OMV-stimulating response to the PQS structure (33, 35) help to explain how the asymmetric distribution of PQS across leaflets may be established and maintained. This model describes a general mechanism by which *P. aeruginosa* (or any organism) might control OMV formation in the absence of external stress. Other models describe OMV biogenesis as a direct response to membrane stress (36–38) or “malfunctions” of peptidoglycan turnover, cell division, or lipid transport (39–41). However, experimental observations of consistent and predictable OMV production by many organisms under a variety of conditions suggest that a mechanism must exist for OMV production independent of the presence of transient membrane or periplasmic stressors.

PQS synthesis proceeds through the condensation of anthranilate with fatty acid to produce 2-heptyl-4-quinolone (HHQ) (42), which is then hydroxylated by the PqsH enzyme to form PQS (32). Based upon protein sequence analysis and substrate requirements (32), these reactions are believed to occur in the cytoplasm. However, the bilayer-couple model predicts that PQS must be transported out of the cell (through an as-yet-unidentified export mechanism) to induce OMV biogenesis. The goal of this study was to uncover whether PQS export plays an important role in the production of OMVs. Examining both laboratory-adapted and clinical strains, we found significant differences in PQS export that correlated strongly with OMV formation. Subcellular fractionation revealed that poor OMV producers had markedly different distributions of PQS between membrane compartments (inner membrane [IM], OM, OMV) than strong OMV producers. Accumulation of PQS in the inner membrane was a hallmark of poor OMV producers, suggesting that low OMV production was a result of inefficient PQS export rather than lack of production. We showed that the membrane distribution of PQS for an individual strain was stable over time but that it could be altered by growth in a different medium, with corresponding alteration in OMV production. This work takes the first look at how PQS membrane distribution affects OMV production and suggests that regulation of PQS localization can serve as a way to modulate secretion of OMV-associated effectors.

## RESULTS

### Extent of PQS export is different between strains of *P. aeruginosa*.

**FIG 1:**
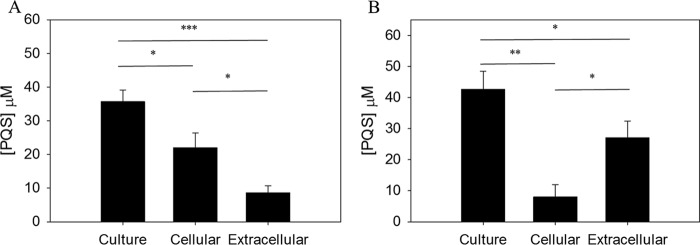
Strain differences affect PQS export. *P. aeruginosa* cultures were grown in 500 ml of LB for 18 h. PQS was extracted with acidified ethyl acetate and analyzed by TLC. Densitometry was used to determine PQS concentrations in whole culture and in the cellular and extracellular compartments in PAO1 (A) and PA14 (B). Error bars represent the standard deviations calculated from three independent experiments. Asterisks indicate statistically significant differences using a two-tailed *t* test. *, *P* ≤ 0.05; **, *P* ≤ 0.01; ***, *P* ≤ 0.001.

Two commonly used laboratory strains, PAO1 and PA14, were grown in Luria-Bertani broth (LB). As expected, the levels of growth of both strains were comparable (see Fig. S1 in the supplemental material). PQS was extracted using acidified ethyl acetate during stationary growth phase (18 h of growth for our 500-ml cultures). This time point was chosen to coincide with maximal PQS production under our conditions (Fig. S2) and is in good agreement with the growth phase corresponding to peak PQS production (43–45) and PqsH expression (29) observed by others. In our PA14 cultures, the majority (78.0% ± 11.1%) of PQS was found to be extracellular, which is consistent with results reported by others using this strain (16) (Fig. 1B). With PAO1, however, we observed an inverse distribution. In this case, 71.2% ± 11.3% of PQS was found to be cell associated, while only 28.8% ± 11.3% was present in the medium supernatant (Fig. 1A). Interestingly, this was not the result of differences in total PQS production (two-tailed *t* test *P* = 0.225) (Fig. 1). However, the striking difference in PQS distribution led to a large difference in the amounts of PQS found in the culture supernatant (two-tailed *P* = 0.011) (Fig. 1). These results uncovered differences in PQS export between two strains, which allowed us to go on to test the correlation between extracellular PQS and OMV production.

10.1128/mBio.01034-17.1 FIG S1  Growth levels are comparable for PAO1 and PA14. Growth curves of *P. aeruginosa* strains PAO1 and PA14 grown in 500 ml of LB. OD_600_ measurements were taken every 2 to 4 h for 36 h. Error bars represent the standard deviations calculated from three independent experiments. Download FIG S1, TIF file, 0.1 MB. Copyright © 2017 Florez et al. 2017 Florez et al. This content is distributed under the terms of the Creative Commons Attribution 4.0 International license.

10.1128/mBio.01034-17.2 FIG S2  PQS production for PAO1 and PA14 grown in 500-ml LB cultures. PQS was extracted with ethyl acetate, analyzed by TLC, and measured over a 22-h time span. Error bars represent the standard deviations calculated from three independent experiments. Download FIG S2, TIF file, 0.1 MB. Copyright © 2017 Florez et al. 2017 Florez et al. This content is distributed under the terms of the Creative Commons Attribution 4.0 International license.

### OMV production parallels extracellular PQS concentrations.

**FIG 2:**
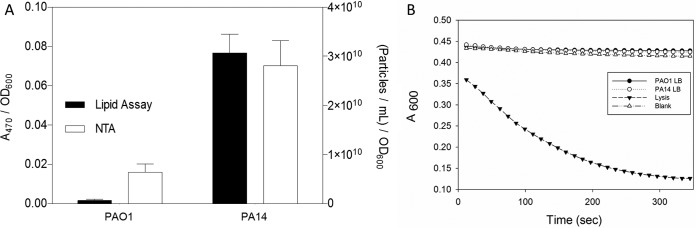
Strain differences in OMV production parallel those in PQS export. (A) OMVs from 500-ml cultures were collected by ultracentrifugation and quantified by measuring lipid content and analyzing particle counts using NTA. Values were normalized to a final culture OD_600_. Error bars represent the standard deviations calculated from three independent experiments. (B) SDH activity in the supernatant was minimal at the time of OMV harvest. The results depicted are representative of three independent experiments.

As a result of the conspicuous strain-dependent effects on the distribution of PQS between cells and the supernatant, it was of interest to test whether differences in extracellular PQS were associated with differences in OMV production. This was done in two ways: nanoparticle-tracking analysis (NTA), carried out using the NanoSight NS30 instrument, and a lipid quantification method (32, 46). NTA and lipid assays were performed on vesicles obtained by ultracentrifugation from culture supernatants of cells grown to stationary phase (18 h, 500-ml cultures) (Fig. S1). Both NTA and lipid analysis showed a positive correlation between extracellular PQS and OMV concentration (Fig. 2A). In order to ensure that the particles or extracellular lipids detected were not the result of cell lysis, succinate dehydrogenase (SDH) assays were performed to test for the presence of IM contamination in the supernatant at the time of harvest. SDH activity was undetectable in the supernatant, confirming that the measured quantities of particles and lipid were representative of bona fide OMV production (Fig. 2B).

### What does it mean for PQS to be cell associated?

Our findings suggested that OMV production was correlated to the amount of PQS exported, not to the total amount of PQS produced. PA14, which is a strong OMV producer, exported a large majority of its PQS to the supernatant, while PAO1, which is a poor OMV producer, retained the majority of its PQS within the cell. This is true despite the fact that both strains produced the same amount of total PQS. This raised an important question: what was the subcellular distribution of PQS that remained associated with the cell, particularly in cells that did not produce large numbers of OMVs? To try to understand poor OMV production in PAO1, we proposed two competing hypotheses: (i) that PQS synthesized within the cell had successfully been translocated to the OM but failed to induce OMV formation and (ii) that synthesized PQS was trapped within the IM and therefore never made contact with the OM to promote OMV formation.

**FIG 3:**
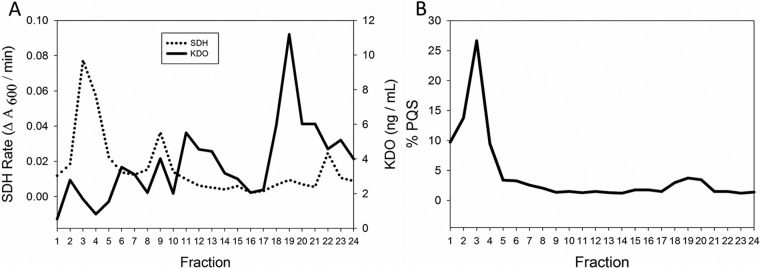
Distribution of cell-associated PQS in PAO1. Fractions (0.5 ml) were obtained from membrane separation of strain PAO1 grown in 500 ml of LB. (A) Peak SDH activity was measured to detect IM fractions, and peak KDO concentration was used to identify OM fractions. (B) PQS was extracted from each fraction. Data are presented as the percentage of cell-associated PQS found in each fraction. The results depicted are representative of three independent experiments.

To discriminate between the two hypotheses, the IM and OM were separated using a discontinuous sucrose density gradient. Samples from the gradient were collected in 0.5-ml fractions that were sequentially removed from the top. Biochemical assays were performed on each fraction to verify effective separation of the two membranes. SDH and 3-deoxy-d-manno-2-octulosonic acid (KDO) were used as IM and OM markers, respectively. SDH activity was localized in the top fractions of the gradient, and KDO concentrations peaked in the bottom fractions (Fig. 3A). Having successfully separated the IM from the OM, we next determined whether PQS was localized to the IM or the OM of cells that were poor exporters. To do this, the same fractions analyzed to confirm membrane separation were assayed for the presence of PQS by first extracting them with acidified ethyl acetate and subsequently quantifying PQS using thin-layer chromatography (TLC) (47). The fractions coinciding with the highest SDH activity (IM marker) also contained the greatest concentration of PQS (Fig. 3B). Minimal PQS was detected throughout the remainder of the gradient except in a small peak that overlapped the OM fractions. These findings indicate that in a poor vesicle-producing strain, such as PAO1, the majority of PQS is found in the IM. A discontinuous sucrose gradient was also performed on the good exporter, PA14, and it was observed that although the majority of cell-associated PQS was also found in the IM (Fig. S3), this comprised a very small percentage of total PQS produced by this strain.

10.1128/mBio.01034-17.3 FIG S3  Distribution of cell-associated PQS in PA14 grown in LB. Fractions (0.5 ml) were obtained from membrane separation of strain PA14 grown in 500 ml of LB. (A) Peak SDH activity was measured to detect IM fractions, and the peak KDO concentration was used to determine OM fractions. (B) PQS was extracted from each fraction. Data are presented as the percentage of cell-associated PQS found in each fraction. The results depicted are representative of three independent experiments. Download FIG S3, TIF file, 0.2 MB. Copyright © 2017 Florez et al. 2017 Florez et al. This content is distributed under the terms of the Creative Commons Attribution 4.0 International license.

### PQS membrane distribution predicts OMV biogenesis in *P. aeruginosa*.

**FIG 4:**
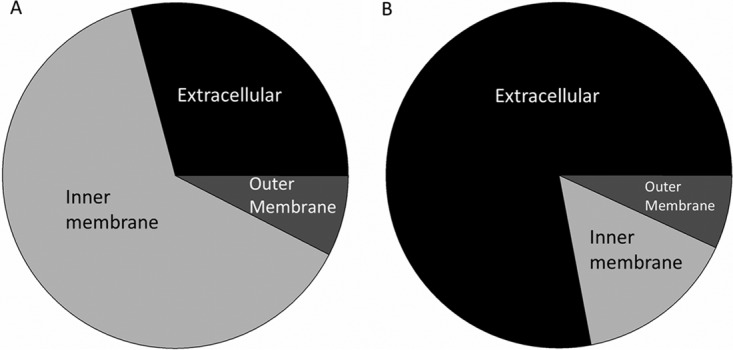
Distributions of PQS in PAO1 and PA14 cultures. PAO1 (A) and PA14 (B) cultures were grown in 500 ml of LB to stationary phase (18 h) and fractionated, and PQS was then extracted and quantified. Pie charts represent the proportion of total PQS found in each compartment. In PAO1 cultures, PQS was distributed as follows: 29.1% extracellular, 63.3% in the IM, and 7.56% in the OM. PA14 cultures showed a strikingly different distribution: 78.0% extracellular, 15.1% in the IM, and 6.90% in the OM.

The distributions of PQS between the IM, OM, and culture supernatant were tested in *P. aeruginosa* strains that naturally produced few (PAO1) or many (PA14) OMVs. Our data reveal that for the low-OMV-production strain, the majority of PQS was found associated with the IM (63.29% ± 1.25%) (Fig. 4A). The OM and supernatant contained 7.56% ± 1.11% and 29.1% ± 0.147% of the total PQS, respectively (Fig. 4A). For the strain producing a high level of OMVs, the IM and OM distributions were 15.1% ± 1.54% and 6.85% ± 2.72%, respectively, and the great majority of PQS was found in the extracellular supernatant (78.0 ± 1.68%) (Fig. 4B). This suggests that movement of PQS out of the IM is critical for OMV production in *P. aeruginosa*.

### Temporal analysis of PQS export and OMV production in PAO1 and PA14.

**FIG 5:**
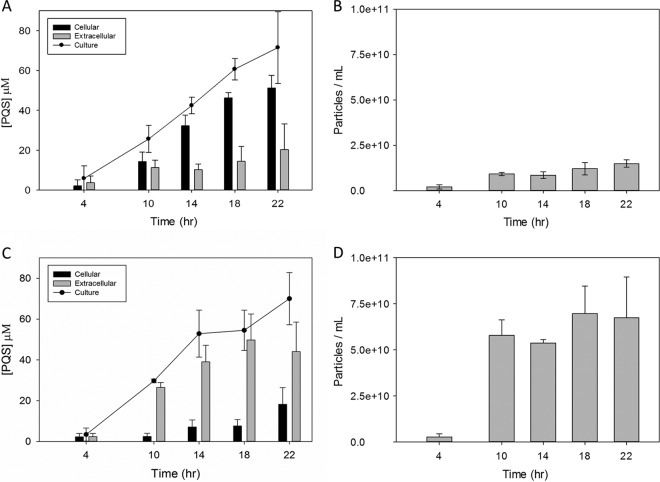
Time course depicting PQS distribution and OMV production in PAO1 and PA14 cultures. Cultures were grown in 500 ml of LB and analyzed over a 22-h time span. PQS was extracted and analyzed by TLC, and densitometry was used to determine PQS concentrations. OMVs were collected by ultracentrifugation and quantified by NTA. (A) PQS distribution in the PAO1 LB culture; (B) OMV production in the PAO1 LB culture; (C) PQS distribution in the PA14 LB culture; (D) OMV production in the PA14 LB culture. Error bars represent the standard deviations calculated from at least three independent experiments.

To determine whether the different PQS export and OMV production phenotypes observed in PAO1 and PA14 were characteristic of each strain or simply an artifact arising from single-time-point sampling, a temporal analysis was performed. PAO1 and PA14 cultures grown in 500 ml of LB showed that very little PQS was produced or exported after 4 h of growth (Fig. 5A and C). This was paralleled by low vesicle production (Fig. 5B and D). At 10 h, both cultures entered late exponential growth phase (Fig. S1) and PQS production dramatically increased (Fig. 5A and C). At this time point, OMV production in PAO1 and PA14 also saw a significant increase (Fig. 5B and D). However, it is important to note that the spike in OMV production was not a consequence of PQS production *per se* but rather of PQS export. This is supported by two main observations. First, OMV production closely correlates with PQS export, not total production. Second, over the 22-h analysis, there was a significant difference between the amounts of PQS exported by the two strains, which directly correlated to the number of OMVs produced by the respective strain. Over time, PAO1 exported significantly less PQS and produced substantially fewer vesicles than PA14, despite producing the same amount of total PQS (Fig. 5).

Based on the pattern of cellular versus extracellular PQS over time, it appears that the difference between strains can be attributed to apparent saturation effects. At 10 h, the ratio of cellular to extracellular PQS in PAO1 cultures was nearly 1:1, but by 18 h, it had changed dramatically to 3:1 (Fig. 5A). During the same timeframe, the raw extracellular PQS concentration remained constant (Fig. 5A). These data suggest that at approximately 10 h, PQS export in PAO1 cultures reached a saturation point. As PQS production continued, it accumulated in the cellular compartment (Fig. 5A). We know from Fig. 4 that the vast majority of this accumulation was in the IM. In PA14, there was no statistically significant difference in extracellular PQS between 10 h and 22 h (Fig. 5C). As PQS production continued to climb, cellular PQS significantly increased, indicating that this strain also underwent cellular PQS accumulation once a maximum extracellular PQS threshold was reached (Fig. 5C).

### Altering PQS distribution affects OMV production in PAO1.

**FIG 6:**
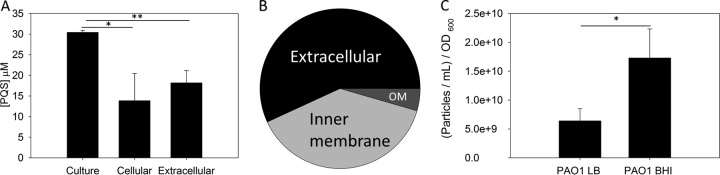
Alteration of PQS distribution in PAO1 grown in BHI. Cultures were grown in 500 ml of BHI for 18 h. (A) PQS was separated by TLC, and densitometry was used to determine PQS concentrations. (B) Membranes were separated by density gradients, and PQS was distributed as follows: 56.9% extracellular, 38.6% in the IM, and 4.48% in the OM. (C) OMVs were collected by ultracentrifugation and quantified using NTA. Values were normalized to the final culture OD_600_. Error bars represent the standard deviation calculated from three independent experiments. Asterisks indicate statistically significant differences using a two-tailed *t* test. *, *P* ≤ 0.05; **, *P* ≤ 0.01.

Our initial analysis showed that PAO1 was a poor PQS exporter and low OMV producer but that PA14 was a good PQS exporter and high OMV producer. In addition, it was demonstrated through a temporal study that PAO1 and PA14 quickly establish strain-specific export equilibria. It was of interest to determine if any conditions could alter these relatively stable PQS export and OMV production phenotypes. We first grew PAO1 in brain heart infusion broth (BHI), a common rich and complex medium that has previously been reported to support strong OMV production by PA14 (16). Remarkably, and in contrast to its growth in LB, PAO1 dramatically changed its PQS distribution when grown in BHI (Fig. 6a). PAO1 grown in BHI exported significantly larger amounts of PQS than PAO1 grown in LB (two-tailed *t* test *P* = 0.0216), despite producing similar amounts of total PQS (two-tailed *t* test *P* = 0.0995). The growth rate of PAO1 was comparable to that of PA14 under these conditions (Fig. S1), indicating that changes in PQS export were not due to growth differences. Membrane separation showed that the overall PQS distribution of PAO1 grown in BHI more resembled that of PA14 than that of PAO1 grown in LB, with the majority of PQS found in the extracellular compartment (57.0% ± 0.13%) and smaller amounts of PQS in the inner and outer membranes (38.5% ± 0.24% and 4.50% ± 0.37%, respectively) (Fig. 6B and S4). NTA was employed to determine if altered PQS distribution increased OMV production under these conditions; indeed, we found that PAO1 grown in BHI produced more vesicles (two-tailed *t* test *P* = 0.0256) (Fig. 6C). SDH activity was undetectable in supernatants harvested from these conditions, ensuring that debris from cell lysis did not influence OMV quantification analyses (Fig. S5).

10.1128/mBio.01034-17.4 FIG S4  Distribution of cell-associated PQS in PAO1 grown in BHI. Fractions (0.5 ml) were obtained from membrane separation of strain PAO1 grown in 500 ml of BHI. (A) Peak SDH activity was measured to detect IM fractions, and the peak KDO concentration was used to determine OM fractions. (B) PQS was extracted from each fraction. Data are presented as the percentage of cell-associated PQS found in each fraction. The results depicted are representative of three independent experiments. Download FIG S4, TIF file, 0.2 MB. Copyright © 2017 Florez et al. 2017 Florez et al. This content is distributed under the terms of the Creative Commons Attribution 4.0 International license.

10.1128/mBio.01034-17.5 FIG S5  SDH activity in the supernatant of PAO1 and PA14 grown in 500 ml of BHI was minimal at the time of OMV harvest. The results depicted are representative of three independent experiments. Download FIG S5, TIF file, 0.1 MB. Copyright © 2017 Florez et al. 2017 Florez et al. This content is distributed under the terms of the Creative Commons Attribution 4.0 International license.

### PQS export positively correlates with OMV formation in lab strains and clinical isolates.

**FIG 7:**
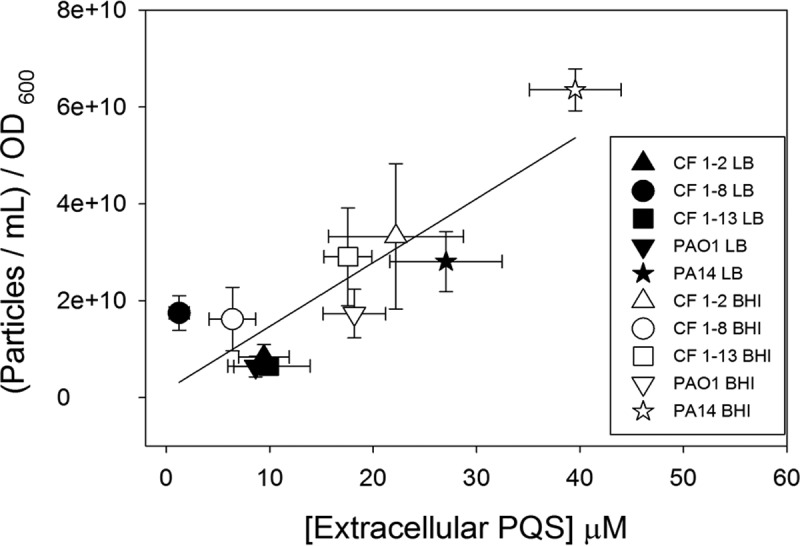
PQS export predicts OMV formation in diverse strains of *P. aeruginosa*. Laboratory strains and clinical isolates were analyzed for PQS export and OMV production. PQS was extracted with ethyl acetate and analyzed by TLC. OMVs were collected by ultracentrifugation and quantified using NTA. Error bars represent the standard deviation calculated from at least three independent experiments. *R*² = 0.756.

The medium-dependent differences in PQS export and OMV production that were observed using PAO1 were also seen for PA14. Growth of PA14 in BHI compared to its growth in LB significantly increased the amount of PQS exported and the number of OMVs produced (Fig. 7). Because we saw this relationship hold between two commonly used laboratory strains, we were interested in testing whether it could be generalized across other strains of *P. aeruginosa*. In addition to PAO1 and PA14, three phenotypically distinct cystic fibrosis (CF) clinical strains (Table 1) were grown in LB and BHI media and analyzed for PQS export and OMV production. By combining the data from all strains, it was determined that PQS export is a strong predictor of OMV production in both laboratory strains and clinical isolates (*R*^2^ = 0.756) (Fig. 7).

**TABLE 1:** Strains used in this study.

Strain	Description	Reference
PAO1	Wild type	55
PA14	Wild type	54
CF 1-2	Classic *P. aeruginosa* strain isolated from a newborn diagnosed with CF	56
CF 1-8	Rough *P. aeruginosa* strain isolated from a newborn diagnosed with CF	56
CF 1-13	Mucoid *P. aeruginosa* strain isolated from a newborn diagnosed with CF	56

## DISCUSSION

Production of outer membrane vesicles is ubiquitous among Gram-negative organisms and has been associated with many important cellular behaviors, ranging from threat avoidance to competition, virulence, and biofilm formation (reviewed in reference 48). Despite this, we know very little about how OMVs are formed. We have taken advantage of a (so far) unique situation in *P. aeruginosa* to propose a mechanistic model of OMV biogenesis. Our model describes preferential intercalation of PQS, a self-produced small molecule, into the outer leaflet of the OM, which induces an interleaflet tension that is relieved through induction of membrane curvature and subsequent OMV release (33). In this study, we investigated whether OMV production in *P. aeruginosa* requires PQS to be exported from the cell.

To examine the relationship between PQS localization and OMV biogenesis, we had to first identify conditions under which the distribution of PQS between compartments was different. Two commonly used lab strains (PAO1 and PA14) were grown in LB, and we found that they produced equal amounts of PQS but that they differed drastically in PQS distribution. In PAO1, the majority of PQS was found to be cell associated, whereas in PA14, the majority of PQS was found to be in the supernatant (Fig. 1). This was followed up by testing whether differences in extracellular PQS concentrations affected OMV production. Using two independent measurement techniques, we found that the level of OMV production aligned with the amount of PQS that was exported into the medium (Fig. 2). This established that OMV production is more closely related to the amount of PQS exported than to the total amount of PQS produced.

We were intrigued by our discovery that a significant amount of PQS remained associated with the cell in the strain with poor OMV production. To determine where the cell-associated PQS resided, we fractionated the subcellular membrane compartments via sucrose density centrifugation. We discovered that for PAO1, the strain with poor PQS export that had diminished OMV production, PQS was overwhelmingly found associated with the IM and that only 29.1% of total PQS was exported to the supernatant (Fig. 4A). Conversely, PA14 exported 78.0% of total PQS to the supernatant and had a correspondingly higher production of OMVs (Fig. 4B). These results suggest that the reason behind the strain difference in OMV production is their varied abilities to move PQS from the IM. Good OMV producers efficiently export PQS from the IM, and poor OMV producers do not.

Our finding that the majority of PQS synthesized by cells with poor OMV production remains localized to the IM was unexpected, and it raised two important questions. (i) Is sequestering the majority of produced PQS in the IM toxic to the cell? (ii) What is the biological benefit of exporting a small percentage of total produced PQS and therefore of making fewer vesicles? First, it has been previously reported that PQS can be toxic at high concentrations (49). However, we showed that PAO1 did not exhibit compromised growth compared to that of PA14, and we could not detect any cell lysis by assaying for succinate dehydrogenase activity in culture supernatants (Fig. 2B and see Fig. S1 in the supplemental material). These results support the conclusion that cell-associated PQS was not detrimental to cells under our conditions and that detected OMVs were not artifactual membrane fragments resulting from cell lysis. IM stability and integrity were also points of interest, because we wondered what effects high concentrations of PQS might have on the IM. Previous biophysical examinations of PQS interactions with lipids uncovered a specific and preferential interaction of PQS with LPS lipid A over phospholipid (34). Because PQS may not have been driven to accumulate in one phospholipid leaflet of the IM over the other phospholipid leaflet, the bilayer-couple model predicts that no induction of membrane curvature should occur. A related phenomenon was demonstrated when we successfully antagonized PQS-induced membrane curvature in surrogate membranes using a second small molecule known to preferentially insert into the opposite membrane leaflet (33). Taken together, these results suggest that PQS accumulation in the IM does not lead to runaway curvature or budding. The biological benefit of exporting a small percentage of total produced PQS, and therefore of making fewer vesicles, is not yet clear. However, previous studies have shown that OMV abundance in the supernatants of *P. aeruginosa* strains correlates with virulence/competition potential. Sabra et al. (31) found that PAO1 supernatants were more effective at killing a hybridoma cell line when they were grown under conditions promoting higher OMV production, and our own group showed that wild-type PA14 cells that were inhibited from releasing OMVs (due to substrate limitation of PqsH) were severely attenuated in clearing *Staphylococcus epidermidis* in a filter disc assay (32). In these same experiments, we saw that *P. aeruginosa* rapidly resumed PQS and OMV production when they were transferred to permissive conditions. Therefore, it is possible that sequestration of PQS in the IM might serve as a way to dampen OMV-mediated virulence or serve as a reservoir to be tapped for rapid OMV production upon encountering more-favorable conditions.

The PQS export system has yet to be identified; however, this work begins to elucidate what factors may impact its function. Under no condition did we find large amounts of PQS associated with the OM. Initially, we had expected that a good OMV producer would display strong localization of PQS to the OM in order that OMV formation could be effectively promoted. Rather, our data suggest that PQS-induced OMV biogenesis is a rapid process that does not require long PQS residence times within the OM. A similar interpretation was made by Baumgarten et al. (50) in studies using strains of *Pseudomonas putida* that expelled exogenously added alkanols in OMVs. Their analysis concluded that the OMV induction response was too rapid to be explained by a transcriptionally controlled response. As an alternative to (or in conjunction with) rapid generation of OMVs, our results may instead provide early evidence for targeted production of OMVs at the sites of PQS export to the OM. If PQS experiences limited lateral diffusion along the plane of the OM, high local concentrations would rapidly accumulate at the site of export, leading to rapid curvature induction and development of OMVs without appreciable buildup of PQS in the bulk OM.

Time course experiments demonstrated that the phenotypic differences observed between PAO1 and PA14 were not an artifact of single-time-point analysis. These experiments revealed that the distinctive PQS distribution phenotypes of PAO1 and PA14 are established early and do not fluctuate greatly with time (Fig. 5). This study further supported the idea that OMV production was driven by PQS export, as there was a continuous positive correlation between PQS export and OMV production over time. An interesting finding was that, for both strains, there appeared to be a point of saturation where net export of PQS severely diminished and cellular accumulation began. The fact that PAO1 saturated at a much lower extracellular PQS concentration than PA14 explains why PAO1 had significantly higher cell-associated PQS than PA14 and significantly lower OMV production. We cannot rule out the possibility that vesicle fusion bringing PQS back to the producer cell (or neighbors) contributed to the observed export plateau, though the consistently small amount of PQS observed in the OM argues against this. If such a phenomenon occurred in our system, it would indicate that the OM residence time is short for PQS entering as well as leaving the cell.

We are very interested in understanding the factors that give rise to differences in PQS export, and this comprises an active area of research in our group. As a first step toward this understanding, we uncovered an effect of growth medium that directly influenced PQS export and OMV biogenesis. Growth in BHI positively affected PQS export, and this positively correlated with an increase in vesicle production (Fig. 6). We hypothesize that nutrient and ion availability in the medium may play a role in this, which would be consistent with the results of previous studies that linked glucose and magnesium levels to OMV production (29, 51). Metabolic flux has also been shown to be important in the production of PQS (52, 53) and may affect export as well. The fact that growth in a different medium was capable of altering PQS membrane distribution within an individual wild-type strain that corresponded to predictable changes in OMV production strongly supports the idea that PQS export is a factor that can be manipulated by cells in order to modulate OMV production.

In two laboratory strains, we observed that increased PQS export had a positive effect on OMV production. Therefore, we were interested to know if this paradigm could be extended to clinical strains. We tested three phenotypically distinct cystic fibrosis (CF) isolates, and they all displayed a direct correlation between exported PQS and OMV production, suggesting that this link is biologically relevant across an array of genetic backgrounds and growth environments (Fig. 7).

The packaging and release of OMVs represents a poorly understood mechanism of secretion that impacts many areas of bacterial physiology and pathogenesis. With this work, we provide the first examination of the distribution of PQS within the cell and the impact of this distribution on its ability to induce OMV biogenesis. We showed that PQS export, rather than PQS production, is the best predictor of OMV biogenesis in both laboratory and clinical strains of *P. aeruginosa* and that cells can alter the amount of PQS that is exported under different conditions to correspondingly modulate OMV production. These findings contribute to a foundational understanding of small-molecule-induced OMV biogenesis in *P. aeruginosa* and provide the framework for more detailed study of PQS export and the mechanisms by which it is regulated.

## MATERIALS AND METHODS

### Strains, growth conditions, and media.

Experiments were carried out using *P. aeruginosa* strains PA14 (54) and PAO1 (55) and CF clinical isolates (56). Cultures were inoculated to an optical density at 600 nm (OD_600_) of 0.01 and were grown for 18 h at 37°C with shaking at 250 rpm. All cultures were grown in 500 ml of either Luria-Bertani broth (LB) or brain heart infusion broth (BHI), unless otherwise stated.

### Whole culture and cellular and extracellular PQS extraction and quantification.

Cultures were centrifuged twice at 15,000 × *g* for 15 min in order to separate cells from the supernatant. Following the second centrifugation, the supernatant was filtered using a 0.45-µm filter. Cell pellets were resuspended in MV buffer (50 mM Tris, 5 mM NaCl, 1 mM MgSO4, pH 7.4) up to their original volume. PQS was extracted from whole culture and from cellular and extracellular components with a 1:1 addition of acidified ethyl acetate (0.1 ml/liter acetic acid). The organic phase was removed and dried under nitrogen gas. Dried samples were resuspended in 100 µl of methanol (Optima grade; Fisher), and 5-µl specimens were spotted onto a straight-phase phosphate-impregnated TLC plate (EM Biosciences), which had been activated for 1 h at 100°C. The mobile phase was 95:5 dichloromethane-methanol. PQS was visualized by intrinsic fluorescence after excitation under long-wave UV light. Digital images were captured and analyzed using the UVP, Inc., Gel Doc-It^2^ imaging system and its densitometry software. Percentages of PQS for the cellular and extracellular compartments were obtained by dividing the individual compartment values by the sum of the cellular and extracellular values.

### OMV isolation.

Cultures were centrifuged twice (15,000 × *g* for 15 min) to pellet cells. Remaining cells were removed from supernatants via filtration through a 0.45-µm filter membrane. Cell-free supernatants were centrifuged at 50,000 rpm for 1.5 h (Thermo Scientific S50-A rotor) to pellet vesicles. Pellets were resuspended in 1-ml MV buffer and quantified by either lipid analysis or NTA.

### Lipid analysis.

OMVs were quantified by lipid content according to previously published protocols (32, 46). Following ultracentrifugation, OMV pellets were resuspended in 1-ml MV buffer. Vesicles were extracted 1:1 with chloroform. The organic layer was removed, combined 1:1 with ammonium ferrothiocyanate solution (23.03 g/liter FeCl_3_·6H_2_O, 30.4 g/liter NH_4_SCN), and mixed thoroughly. The organic layer was removed and analyzed for absorbance at 470 nm. Absorbance values were normalized to the OD_600_ of the extracted culture.

### Nanoparticle tracking analysis.

OMVs were also quantified by direct nanoparticle counting. Pelleted vesicles were resuspended in 1 ml of MV buffer and diluted 1:20 to 1:100 in order to obtain 20 to 100 particles per frame (as per the manufacturer’s instructions). Vesicle size and concentration were analyzed using a NanoSight NS300 system and its corresponding Nanoparticle Tracking and Analysis software (NTA 3.1). A camera level of 12 and a gain of 1 were manually programed to optimize data collection. Each sample was analyzed three times for 30 s at 25°C using different fields of view. Frame sequences were analyzed under manual particle detection and tracking parameters (screen gain of 10 and detection threshold of 23). Values were normalized to the OD_600_ of the extracted culture.

### Lysis assay.

Supernatants were separated from cells by centrifugation (15,000 × *g* for 15 min) and filter sterilized using a 0.45-µm filter. Control samples were lysed via sonication. SDH activity (described below) in culture supernatants and that in control samples were measured and compared in order to ensure that cell lysis was not significant at the time of harvest (18 h).

### Inner and outer membrane separation.

The OM was separated from the IM using a procedure adapted from the work of Hancock and Nikaido (57). Cells were harvested by centrifugation (15,000 × *g* for 15 min) and washed with 30 mM Tris buffer (pH 8). Cell pellets were resuspended in 30 ml of 20% (wt/vol) sucrose in 30 mM Tris buffer (pH 8). Cells were lysed via sonication. Prior to sonication, HALT protease inhibitor cocktail (Thermo, Fisher Scientific) was added as a preventive measure. Unlysed cells were removed by centrifugation (10,000 × *g* for 10 min), and the supernatant was centrifuged at 100,000 × *g* (Thermo Scientific S50-A rotor) for 1 h. Pelleted cell membranes were resuspended in 1 ml of 20% (wt/vol) sucrose in 30 mM Tris buffer (pH 8). One milliliter of cell membranes resuspended in 20% (wt/vol) sucrose was layered on top of a sucrose density gradient consisting of 8 ml of 60% (wt/vol) sucrose and 3 ml of 70% (wt/vol) sucrose. The gradient was centrifuged for 18 h at 32,700 rpm (Beckman SW41-Ti rotor), and the sample was collected from the top in 0.5-ml fractions. All fractions were diluted with 30 mM Tris buffer (pH 8) and centrifuged at 100,000 × *g* in a rotor (Thermo Scientific model S120AT2) in order to remove any remaining sucrose. The pellets were resuspended in 0.5 ml of 30 mM Tris buffer (pH 8) and stored at −70°C.

### Succinate dehydrogenase activity assay.

The SDH assay was a modification of the method of Kasahara and Anraku (58). All fractions collected from the sucrose density gradient were tested for SDH activity. Reactions were carried out in a 96-well plate (Falcon) in a total volume of 200 µl and were comprised of 50 mM Tris-HCl (pH 8.0), 4 mM potassium cyanide (KCN), 0.04 mM 2,6-dichlorophenolindophenol (DCPIP), 0.2 mM phenazine methosulfate (PMS), 40 mM disodium succinate, and 1 µl of sample. The reaction mixture without sample, DCPIP, or PMS was incubated for 5 min at room temperature. Following this incubation period, the sample (enzyme) was added to the mixture and allowed to acclimate for 5 min. Lastly, DCPIP and PMS were added in this order to initiate the reaction. SDH activity was quantified by measuring the absorbance at 600 nm over time at 25°C (Tecan Infinite M-200 Pro).

### KDO assay.

This method was modified from the work of Osborn et al. (59). All fractions from the sucrose density gradient were tested for the presence of 3-deoxy-d-manno-2-octulosonic acid (KDO). KDO standards of 0, 4, 8, 16, and 20 µg/ml were prepared from a 100-mg/liter KDO stock solution. Twenty-five microliters of the standard or sample was utilized for each reaction. Following a 1:1 addition of 0.5 M H_2_SO_4_, samples were boiled for 8 min at 100°C to release KDO sugars. Samples were allowed to cool for 10 min at room temperature, after which 25 µl of 0.1 M periodic acid was added and samples were vortexed and incubated at 25°C for 10 min. Next, 100 µl of 0.2 M sodium arsenate in 0.5 M HCl was added. This was followed by vortexing, the addition of 400 µl of 0.6% freshly prepared thiobarbituric acid, and boiling for 10 min. Samples were cooled at room temperature for 35 min. Following this, 750 µl of acidified *n*-butanol was added to each sample, the organic layer was recovered, and absorbance was measured at 552 nm and 509 nm. Measurements of absorbance at 509 nm were subtracted from the 552-nm measurements, and a standard curve was used to determine KDO concentration.

### PQS extraction for sucrose gradient fractions.

PQS was extracted from 0.5-ml fractions as previously described. The organic phase was removed and dried under nitrogen gas. Dried samples were resuspended in 20 µl of methanol (Optima grade; Fisher). PQS was separated and quantified as previously described.

### Time course analysis.

PAO1 and PA14 cultures grown in 500 ml of LB were analyzed for PQS distribution and OMV production over a 22-h time period. Cellular and extracellular PQS concentrations were examined at 4 h, 10 h, 14 h, and 22 h from 2 ml as previously described above. Total PQS production at each time point was calculated by adding cellular and extracellular PQS concentrations. OMV production for each time point was analyzed by NTA from vesicle pellets harvested as previously described from 20 ml of filtered (with a 0.45-µm pore size) supernatant.

### Clinical isolate analysis.

PQS concentrations in culture and in cellular and extracellular fractions were determined from 30-ml cultures and harvested after 12 h of growth. OMVs were extracted (as previously described) and quantified by NTA.
