# Supplementary figures and images for: Membrane Distribution of the Pseudomonas Quinolone Signal Modulates Outer Membrane Vesicle Production in Pseudomonas aeruginosa
Source: mBio. 2017 Aug 8;8(4):e01034-17. doi: 10.1128/mBio.01034-17 (PMC5550756; doi:10.1128/mBio.01034-17)

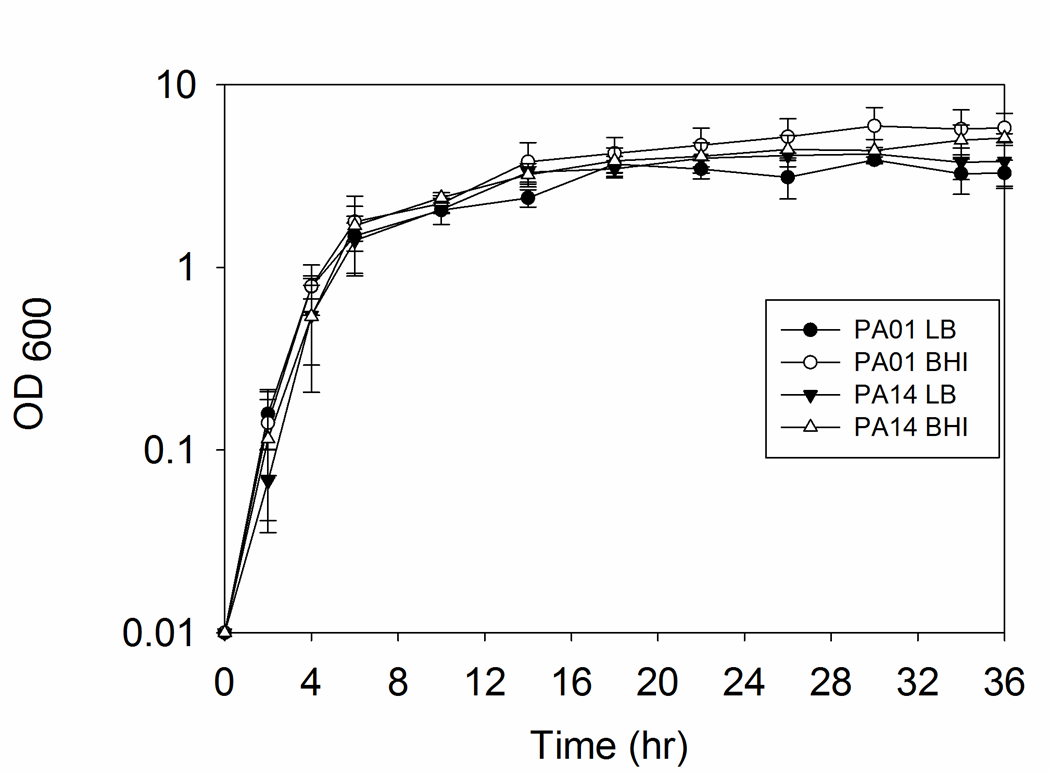

Supplement: FIG S1 [file mbo004173418sf1.tif]

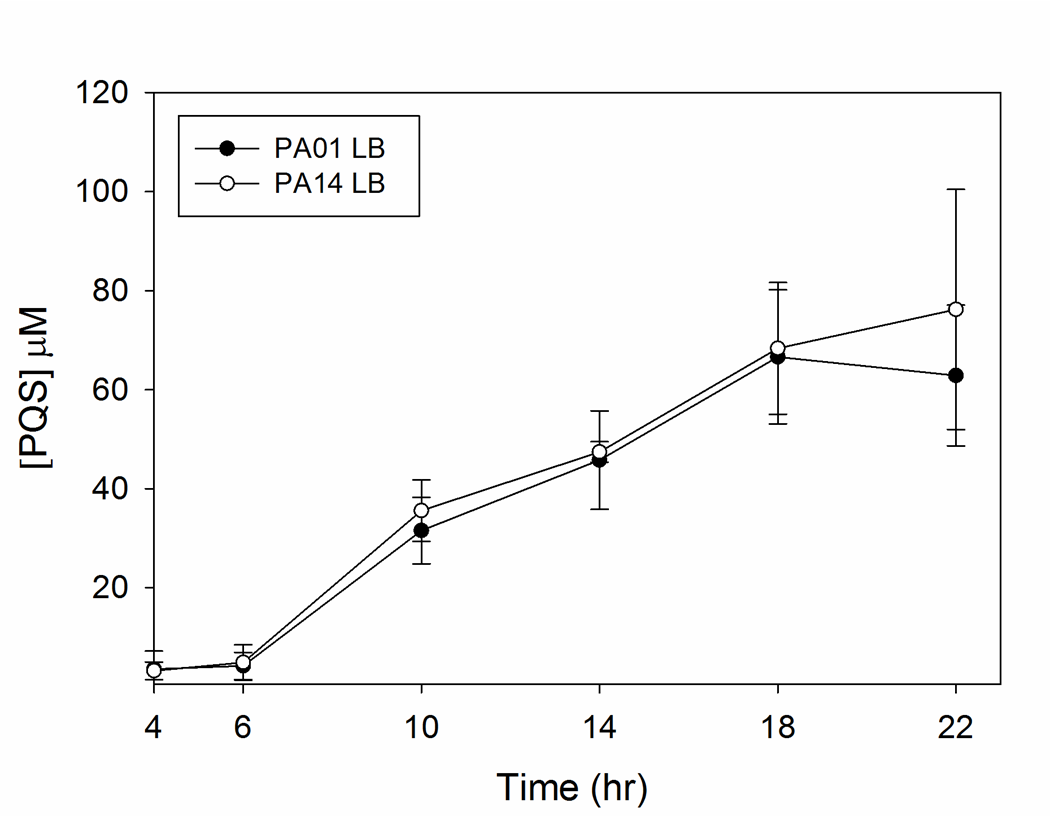

Supplement: FIG S2 [file mbo004173418sf2.tif]

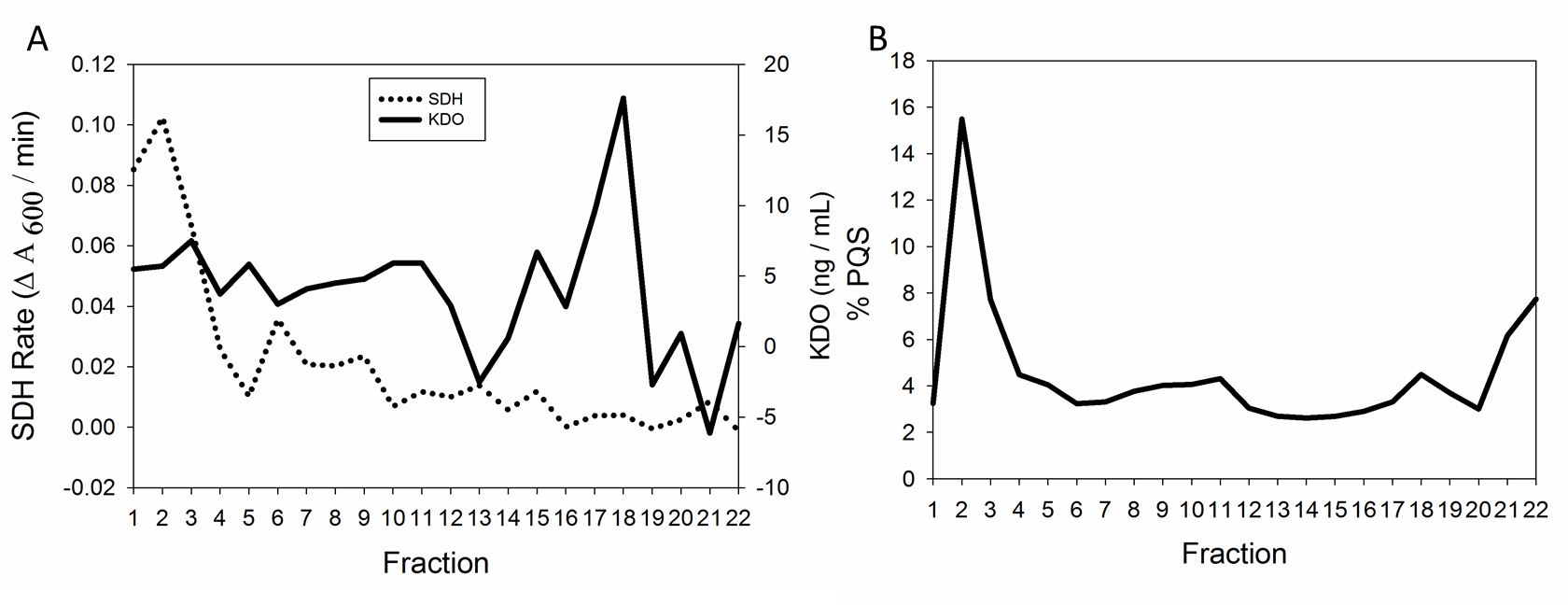

Supplement: FIG S3 [file mbo004173418sf3.tif]

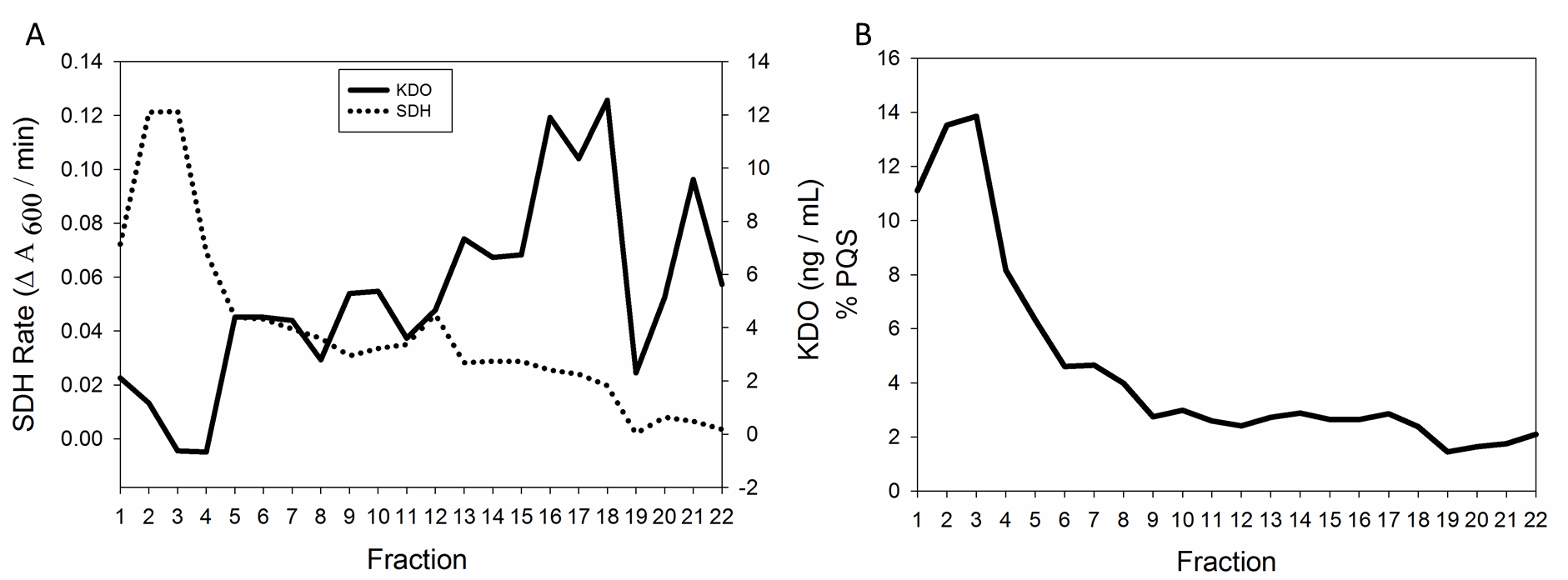

Supplement: FIG S4 [file mbo004173418sf4.tif]

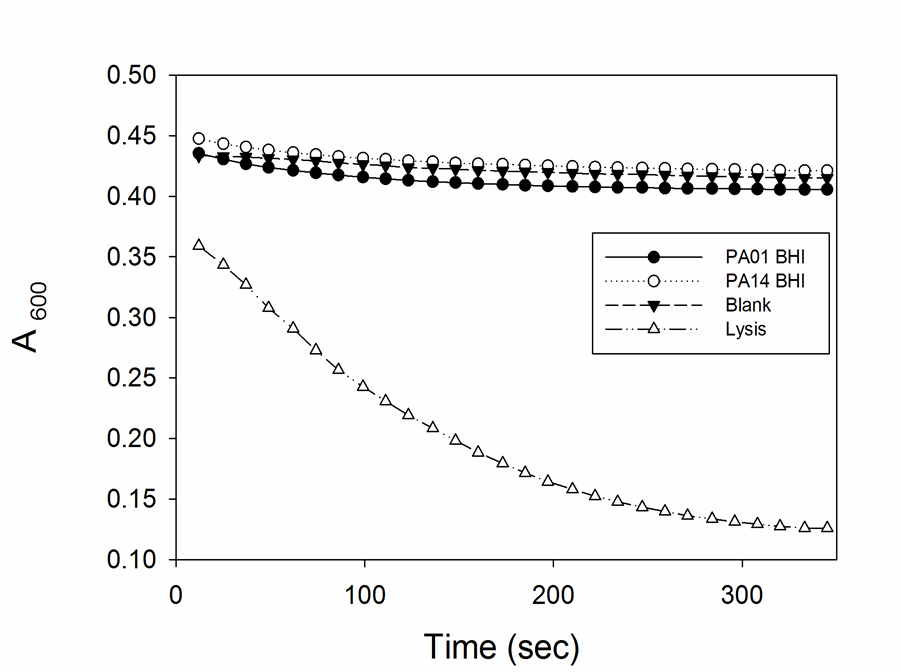

Supplement: FIG S5 [file mbo004173418sf5.tif]
